# Novel Bat-borne Hantavirus, Vietnam

**DOI:** 10.3201/eid1907.121549

**Published:** 2013-07

**Authors:** Satoru Arai, Son Truong Nguyen, Bazartseren Boldgiv, Dai Fukui, Kazuko Araki, Can Ngoc Dang, Satoshi D. Ohdachi, Nghia Xuan Nguyen, Tien Duc Pham, Bazartseren Boldbaatar, Hiroshi Satoh, Yasuhiro Yoshikawa, Shigeru Morikawa, Keiko Tanaka-Taya, Richard Yanagihara, Kazunori Oishi

**Affiliations:** National Institute of Infectious Diseases, Tokyo, Japan (S. Arai, K. Araki, H. Satoh, S. Morikawa, K. Tanaka-Taya, K. Oishi);; Institute of Ecology and Biological Resources, Hanoi, Vietnam (S.T. Nguyen, C.N. Dang, N.X. Nguyen, T.D. Pham);; National University of Mongolia, Ulaanbaatar, Mongolia (B. Boldgiv);; National Institute of Biological Resources, Seoul, South Korea (D. Fukui);; Hokkaido University, Sapporo, Japan (S.D. Ohdachi);; Institute of Veterinary Medicine, Ulaanbaatar (B. Boldbaatar); Chiba Institute of Science, Chiba, Japan (Y. Yoshikawa);; University of Hawaii at Manoa, Honolulu, Hawaii, USA (R. Yanagihara)

**Keywords:** Hantavirus, bats, phylogeny, Vietnam, viruses

**To the Editor**: Compelling evidence of genetically distinct hantaviruses (family *Bunyaviridae*) in multiple species of shrews and moles (order Soricomorpha, families Soricidae and Talpidae) across 4 continents (*1*–*7*) suggests that soricomorphs, rather than rodents (order Rodentia, families *Muridae* and *Cricetidae*), might be the primordial hosts (*6*,*7*). Recently, the host range of hantaviruses has been further expanded by the discovery that insectivorous bats (order Chiroptera) also serve as reservoirs (*8*,*9*). Conjecturing that Mouyassué virus in the banana pipistrelle (*Neoromicia nanus*) in Côte d’Ivoire (*8*) and Magboi virus (MGBV) in the hairy split-faced bat (*Nycteris hispida*) in Sierra Leone (*9*) represent a much broader geographic distribution of bat-borne hantaviruses, we analyzed tissues from bats captured in Mongolia and Vietnam.

Total RNA was extracted from 51 lung tissues, collected in RNAlater Stabilization Reagent (QIAGEN, Valencia, CA, USA), from insectivorous bats, representing 7 genera and 12 species, captured in Mongolia and Vietnam. cDNA was then prepared by using PrimeScript II 1st strand cDNA Synthesis Kit (Takara Bio, Otsu, Shiga, Japan) for reverse transcription PCR (RT-PCR), and using oligonucleotide primers previously designed for amplification of soricid- and talpid-borne hantaviruses (*1*–*7*).

A novel hantavirus, designated Xuan Son virus (XSV), was detected in 1 of 5 Pomona roundleaf bats (*Hipposideros pomona*) by using a heminested large (L)–segment primer set (outer: HNL-2111F, 5′-CARTCWACWGTIGGIGCIAGTGG-3′, and HAN-L-R1, 5′-AACCADTCWGTYCCRTCATC-3′; inner: HNL-2111F and HAN-L-R2, 5′-GCRTCRTCWGARTGRTGDGCAA-3′) and a nested small (S)–segment primer set (outer: OSM55F, 5′-TAGTAGTAGACTCC-3′, and XSV-S6R, 5′-AGITCIGGRTCCATRTCRTCICC-3′; inner: Cro-2F, 5′-AGYCCIGTIATGRGWGTIRTYGG-3′, and JJUVS-1233R, 5′-TCACCMAGRTGRAAGTGRTCIAC-3. The bat was captured during July 2012 in Xuan Son National Park, a nature reserve in Thanh Sơn District, Phu Tho Province, ≈100 km west of Hanoi (21°07′26.75′′N, 104°57′29.98′′E).

For confirmation, RNA extraction and RT-PCR were performed independently in a laboratory in which hantaviruses had never been handled. After initial detection, the L-segment sequence was extended by using another primer set (PHL-173F: 5′-GATWAAGCATGAYTGGTCTGA-3′; and TNL-5084R: 5′-GATCCTGAARTACAATGTGCTGG-3′). To calculate the number of virus copies in tissues by real-time RT-PCR, we used a virus-specific primer set (XSV-F: 5′-GTTGCACAGCTTGGTATTGG-3′; and XSV-R: 5′-TTAGCACCCAAACCTCCAAG-3′) and probe (XSV-Probe: 5′-ACAGCTCCTGGCATGGTAAATTCTCC-3′).

Pairwise alignment and comparison (with ClustalW, www.clustal.org) of a 4,582-nt (1,527 aa) region of the RNA-dependent RNA polymerase–encoding L segment indicated sequence similarities of 71.4%–71.5% and 75.9%–78.7% at the nucleotide and amino acid levels, respectively, between XSV and Mouyassué virus and MGBV. Sequence analysis of a 499-nt (166 aa) region of the nucleocapsid-encoding S segment showed that XSV differed by 42.8%–58.3% from representative hantaviruses harbored by rodents and most soricomorphs. XSV sequences were identical in lung, liver, kidney, and spleen; and the highest number of virus copies (7.6 × 10^1^) was in lung tissue, determined by real-time RT-PCR. No additional hantavirus-infected Pomona roundleaf bats were found by RT-PCR that used XSV-specific primers.

Phylogenetic analyses was performed with maximum-likelihood and Bayesian methods, and we used the GTR+I+Γ model of evolution, as selected by the hierarchical likelihood-ratio test in MrModeltest version 2.3 and jModelTest version 0.1 (*10*), partitioned by codon position. Results indicated 4 distinct phylogroups, with XSV sharing a common ancestry with MGBV (Figure). Similar topologies, supported by high bootstrap (>70%) and posterior node (>0.70) probabilities, were consistently derived when various algorithms and different taxa and combinations of taxa were used. Moreover, as we reported previously, the incongruence between some hantaviruses and their reservoir hosts might be indicative of host-switching events (*5*–*7*).

**Figure:**
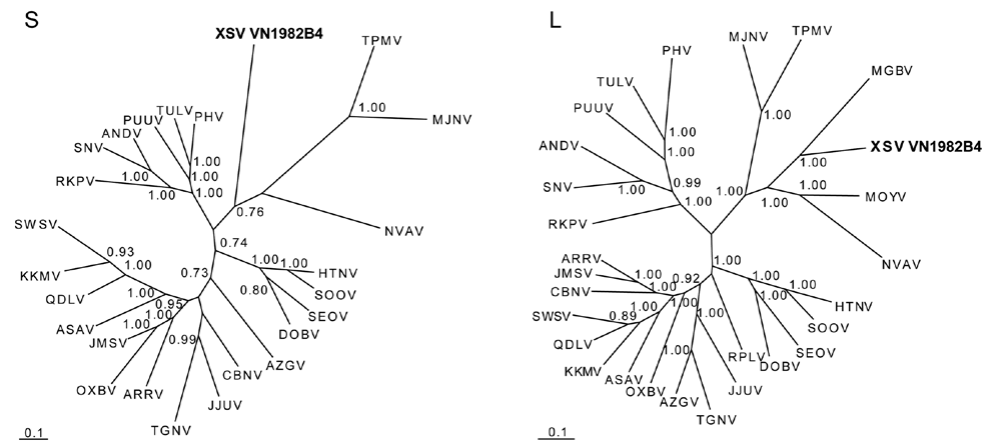
Phylogenetic trees, based on 499-nt and 4,582-nt regions of the small (S) and large (L) genomic segments, respectively, of Xuan Son virus (XSV VN1982B4) (GenBank accession nos. S: KC688335, L: JX912953), generated by the maximum-likelihood and Bayesian Markov chain Monte Carlo estimation methods, under the GTR+I+Γ model of evolution. Because tree topologies were similar when RAxML and MrBayes were used, the tree generated by MrBayes was displayed. The phylogenetic position of XSV is shown in relation to chiropteran-borne hantaviruses, Mouyassué virus ([MOYV] JQ287716) from the banana pipistrelle and Magboi virus ([MGBV] JN037851) from the hairy slit-faced bat. The taxonomic identity of the XSV-infected Pomona roundleaf bat was confirmed by mitochondrial DNA analysis (GenBank accession no. JX912954). The numbers at each node are Bayesian posterior probabilities (>0.7), and the scale bars indicate nucleotide substitutions per site. **Boldface** indicates the Xuan Son virus detected in Pomona roundleaf bat, Vietnam. Representative soricomorph-borne hantaviruses include Thottapalayam virus ([TPMV] AY526097, EU001330) from the Asian house shrew; Imjin virus ([MJNV] EF641804, EF641806) from the Ussuri white-toothed shrew; Jeju virus ([JJUV] HQ663933, HQ663935) from the Asian lesser white-toothed shrew; Tanganya virus ([TGNV] EF050455, EF050454) from the Therese’s shrew; Azagny virus ([AZGV] JF276226, JF276228) from the West African pygmy shrew; Cao Bang virus ([CBNV] EF543524, EF543525) from the Chinese mole shrew; Ash River virus ([ARRV] EF650086, EF619961) from the masked shrew; Jemez Springs virus ([JMSV] FJ593499, FJ593501) from the dusky shrew; Seewis virus ([SWSV] EF636024, EF636026) from the Eurasian common shrew; Kenkeme virus ([KKMV] GQ306148, GQ306150) from the flat-skulled shrew; Qiandao Lake virus ([QDLV] GU566023, GU566021) from the stripe-backed shrew; Camp Ripley virus ([RPLV] EF540771) from the northern short-tailed shrew; Asama virus ([ASAV] EU929072, EU929078) from the Japanese shrew mole; Oxbow virus ([OXBV] FJ539166, FJ593497) from the American shrew mole; Rockport virus ([RKPV] HM015223, HM015221) from the eastern mole; and Nova virus ([NVAV] FJ539168, FJ593498) from the European common mole. Also shown are representative rodent-borne hantaviruses, including Hantaan virus ([HTNV] NC_005218, NC_005222), Soochong virus ([SOOV] AY675349, DQ562292), Dobrava-Belgrade virus ([DOBV] NC_005233, NC_005235), Seoul virus ([SEOV] NC_005236, NC_005238), Tula virus ([TULV] NC_005227, NC_005226), Puumala virus ([PUUV] NC_005224, NC_005225), Prospect Hill virus ([PHV] Z49098, EF646763), Andes virus ([ANDV] NC_003466, NC_003468), and Sin Nombre virus ([SNV] NC_005216, NC_005217).

The striking sequence divergence of XSV presented considerable challenges for designing suitable primers for RT-PCR and sequencing. Also, sequencing efforts were constrained by the limited availability of tissues and concurrent virus isolation attempts. Consequently, we were unable to obtain the full-length sequence of XSV. Similarly, the inability to detect hantavirus RNA in tissues from other species of bats in this study might be attributed to several factors, including the highly focal nature of hantavirus infection, small sample sizes of bats of any given species, primer mismatches, and suboptimal cycling conditions.

Bats of the genus *Hipposideros,* family Hipposideridae, are among the most speciose insectivorous bats; ≈70 species are distributed across Africa, Europe, Asia, and Australia. Pomona roundleaf bats are frequently found in or near limestone or sandstone caves. Their colony sizes vary from few to many hundreds of individuals. The vast geographic distribution of the Pomona roundleaf bat throughout Vietnam and in Bangladesh, Cambodia, China, India, Laos, Malaysia, Myanmar, Nepal, and Thailand, provides opportunities to ascertain the genetic diversity and phylogeography of XSV and XSV-related hantaviruses. In this regard, although hantavirus RNA was not detected in archival tissues from bats of ≈20 genera, including several other *Hipposideros* species (*8*,*9*), many more genetically divergent hantavirus species are probably harbored by insectivorous bats. Not all orphan viruses warrant intensive study at the time of their discovery. However, insights into the ecology and transmission dynamics of newfound bat-borne hantaviruses might prepare us to more rapidly diagnose future outbreaks caused by emerging hantaviruses.
